# Trends in volume-regulated anion channel (VRAC) research: visualization and bibliometric analysis from 2014 to 2022

**DOI:** 10.3389/fphar.2023.1234885

**Published:** 2023-07-19

**Authors:** Tianbao Liu, Yin Li, Dawei Wang, Tobias Stauber, Jiajun Zhao

**Affiliations:** ^1^ Key Laboratory of Endocrine Glucose & Lipids Metabolism and Brain Aging, Ministry of Education, Department of Endocrinology, Shandong Provincial Hospital Affiliated to Shandong First Medical University, Jinan, Shandong, China; ^2^ Shandong Key Laboratory of Endocrinology and Lipid Metabolism, Jinan, Shandong, China; ^3^ Shandong Institute of Endocrine and Metabolic Disease, Jinan, Shandong, China; ^4^ Department of Oncology, Shandong Provincial Hospital Affiliated to Shandong First Medical University, Shandong Provincial Hospital, Jinan, Shandong, China; ^5^ Department of Endocrinology, Shandong Provincial Hospital, Shandong University, Jinan, Shandong, China; ^6^ Institute for Molecular Medicine, MSH Medical School Hamburg, Hamburg, Germany

**Keywords:** volume-regulated anion channel (VRAC), LRRC8, SWELL1, research direction, bibliometric analysis

## Abstract

**Objective:** In this study, we utilized bibliometric methods to assess the worldwide scientific output and identify hotspots related to the research on the volume-regulated anion channel (VRAC) from 2014 to 2022.

**Methods:** From Web of Science, we obtained studies related to VRAC published from 2014 to 2022. To analyzed the data, we utilized VOSviewer, a tool for visualizing network, to create networks based on the collaboration between countries, institutions, and authors. Additionally, we performed an analysis of journal co-citation, document citation, and co-occurrence of keywords. Furthermore, we employed CiteSpace (6.1. R6 Advanced) to analyzed keywords and co-cited references with the strongest burst.

**Results:** The final analysis included a total of 278 related articles and reviews, covering the period from 2014 to 2022. The United States emerged as the leading country contributing to this field, while the University of Copenhagen stood out as the most prominent institution. The author with most publications and most citations was Thomas J. Jentsch. Among the cited references, the article by Voss et al. published in Science (2014) gained significant attention for its identification of LRRC8 heteromers as a crucial component of the volume-regulated anion channel VRAC. Pflügers Archiv European Journal of Physiology and Journal of Physiology-London were the leading journals in terms of the quantity of associated articles and citations. Through the analysis of keyword co-occurrence, it was discovered that VRAC is involved in various physiological processes including cell growth, migration, apoptosis, swelling, and myogenesis, as well as anion and organic osmolyte transport including chloride, taurine, glutamate and ATP. VRAC is also associated with related ion channels such as TMEM16A, TMEM16F, pannexin, and CFTR, and associated with various diseases including epilepsy, leukodystrophy, atherosclerosis, hypertension, cerebral edema, stroke, and different types of cancer including gastric cancer, glioblastoma and hepatocellular carcinoma. Furthermore, VRAC is involved in anti-tumor drug resistance by regulating the uptake of platinum-based drugs and temozolomide. Additionally, VRAC has been studied in the context of pharmacology involving DCPIB and flavonoids.

**Conclusion:** The aim of this bibliometric analysis is to provide an overall perspective for research on VRAC. VRAC has become a topic of increasing interest, and our analysis shows that it continues to be a prominent area. This study offers insights into the investigation of VRAC channel and may guide researchers in identifying new directions for future research.

## 1 Introduction

The ability of cells to modify their volume in reaction to osmotic disturbances is crucial for fundamental physiological processes like growth, migration, and apoptosis (Lang et al., 1998; Hoffmann et al., 2009). Transport proteins facilitate the alterations in cellular volume by allowing the influx or efflux of inorganic ions and larger organic osmolytes, which is accompanied by a flow of water driven by osmosis. Among these regulatory mechanisms, cellular volume alterations regulate various anion and cation channels (Okada et al., 2019; Strange et al., 2019), including the volume-regulated anion channel (VRAC). VRAC is triggered in response to vertebrate cell swelling and plays a critical role in the regulatory volume decrease (RVD) through facilitating chloride and organic osmolytes efflux (Stauber, 2015; Jentsch, 2016; Pedersen et al., 2016; Strange et al., 2019).

VRAC channel has fascinated researchers since its first electrophysiological description in the 1980s (Cahalan and Lewis, 1988; Hazama and Okada, 1988). Numerous laboratories have characterized the biophysical properties of VRAC channel (Strange et al., 1996; Nilius et al., 1997), including its mild outward rectification, which led to its alternative name, volume-sensitive outwardly rectifying channel (VSOR). These investigations have highlighted the crucial role of VRAC in controlling cell volume and its potential involvement in various physiological processes. However, the field faced significant challenge due to perplexity and disagreement surrounding the VRAC’s molecular identity, impeding progress for decades. A significant breakthrough occurred in 2014 when it was demonstrated that Leucine-Rich Repeat-Containing 8A (LRRC8A, also referred to as SWELL1), was an indispensable component of VRAC (Qiu et al., 2014; Voss et al., 2014). There are five members in the LRRC8 protein family, namely, LRRC8A-LRRC8E. VRAC is a heteromeric complex consist of the necessary subunit LRRC8A and at least one of the remaining LRRC8 proteins (LRRC8B to LRRC8E) (Voss et al., 2014; Gaitán-Peñas et al., 2016; Syeda et al., 2016).

The molecular identification of in 2014 set solid ground for its molecular biological investigation and has attracted attention from further researchers in various fields. In subsequent years, significant advancements have been achieved in comprehending the connections between structure and function of VRAC C (König and Stauber, 2019; Kasuya and Nureki, 2022; Sawicka and Dutzler, 2022), along with a plethora of studies investigating the physiological roles of VRAC. These studies have confirmed the participation of VRAC in the regulation of cell volume and its contribution to various associated processes, including proliferation, migration and apoptosis (Osei-Owusu et al., 2018; Chen et al., 2019b). Furthermore, VRAC is crucial for various further processes beyond its role in osmotic volume regulation. This is partly because it not only transports inorganic anions but also organic osmolytes, such as taurine, amino acids, ATP, cGAMP, and xenobiotics like specific antibiotics, cytostatics, and dyes (Hyzinski-García et al., 2014; Lee et al., 2014; Qiu et al., 2014; Voss et al., 2014; Planells-Cases et al., 2015; Gaitán-Peñas et al., 2016; Lutter et al., 2017; Zhou C. et al., 2020; Lahey et al., 2020; Model et al., 2022). Several studies have highlighted the role of VRAC in glucose sensing and insulin signaling (Zhang et al., 2017; Kang et al., 2018; Stuhlmann et al., 2018), myoblast differentiation (Chen et al., 2019a; Chen et al., 2020; Kumar et al., 2020), sperm development (Lück et al., 2018), immune response through cGAMP signaling (Zhou C. et al., 2020; Lahey et al., 2020; Concepcion et al., 2022), NLRP3 activation (Daniels et al., 2016; Green et al., 2020), maintenance of vascular function (Alghanem et al., 2021), and tumor drug resistance (Planells-Cases et al., 2015; Sørensen et al., 2016). Moreover, VRAC has been associated with disease states including agammaglobulinemia (Sawada et al., 2003), male sterility (Bao et al., 2018), type 2 diabetes (Gunasekar et al., 2022), stroke (Yang et al., 2019b; Zhou J. J. et al., 2020), and diverse types of cancer (Zhang et al., 2018; Konishi et al., 2019; Lu et al., 2019; Kurashima et al., 2021). These studies collectively underscore the important role of VRAC in human (patho)-physiology. Despite the growing body of research in the VRAC field, there has been a lack of systematic investigation into the topics and characteristics of most-cited articles regarding the VRAC channel.

Bibliometric studies plays a crucial role in assessing the productivity of institutions, countries, authors, and the frequency of keywords, enabling the exploration of research frontiers in specific fields. Through bibliometric analysis, researchers can gain insight into the current state and developmental trends of research area and offer valuable directions and ideas for future investigations. Among widely utilized bibliometric visualization tools, VOSviewer and CiteSpace have emerged as popular choices for data analysis. Despite the rapid development and multidisciplinary nature of research on the VRAC channel following its molecular identification in 2014, no bibliometric analyses concerning the trends of VRAC research have been conducted to date. In this study, we gathered academic papers on the VRAC channel from 2014 to 2022, by employing bibliometrics and visual analysis methods to investigate the frontiers in the field of VRAC research, with the aim of providing researchers with valuable guidance and insights for their work in this area.

## 2 Methods

### 2.1 Data collection and searching strategy

We obtained the data for our analysis from the Science Citation Index Expanded (SCI- expanded), which is part of the Web of Science Core Collection (WoSCC) database. The data retrieval was performed on 22 March 2023, covering the timespan from 2014 to 2022. To conduct the search, the following keywords were entered into the database: TS = (volume regulated anion channel* OR volume regulatory anion channel* OR LRRC8* OR SWELL1*). All literature retrieval and record downloads were collected in a single day (22 March 2023) to eliminate any potential deviation resulting from daily updates to the database. As this study involved the use of secondary data directly from the database, ethical approval was not required.

### 2.2 Data analysis and visualization

We utilized the WoS Literature Analysis Report online platform to conduct a comprehensive analysis of various publication characteristics. These characteristics included the distribution of publications across different country/region, institutional affiliations, journals, authors, annual publication trends, and citation counts. We gathered the information on the journal impact factors by accessing the 2021 Journal Citation Reports (JCR) provided by Clarivate Analytics in Philadelphia, United States.

VOSviewer (Version 1.6.19), a software created by Leiden University in the Netherlands, was utilized to build networks and visually represent the connections in the VRAC research domain. This tool enabled the examination of co-authorship patterns among countries, institutions, and authors, as well as the analysis of co-citation journals and references, citation patterns of documents, and co-occurrence patterns of keywords. The co-occurrence analysis included keywords that appeared at least twice were included in three visualizations: network, overlay and density. Through these visualizations allowed to recognize significant terms in the field of VRAC research. Furthermore, CiteSpace, a commonly used tool for analyzing research hotspots/Frontier, and future trends, was employed for conducting keyword and co-cited reference burst analysis (CiteSpace 6.1. R6 Advanced).

## 3 Results

### 3.1 Overall details and yearly publication production

**FIGURE 1:**
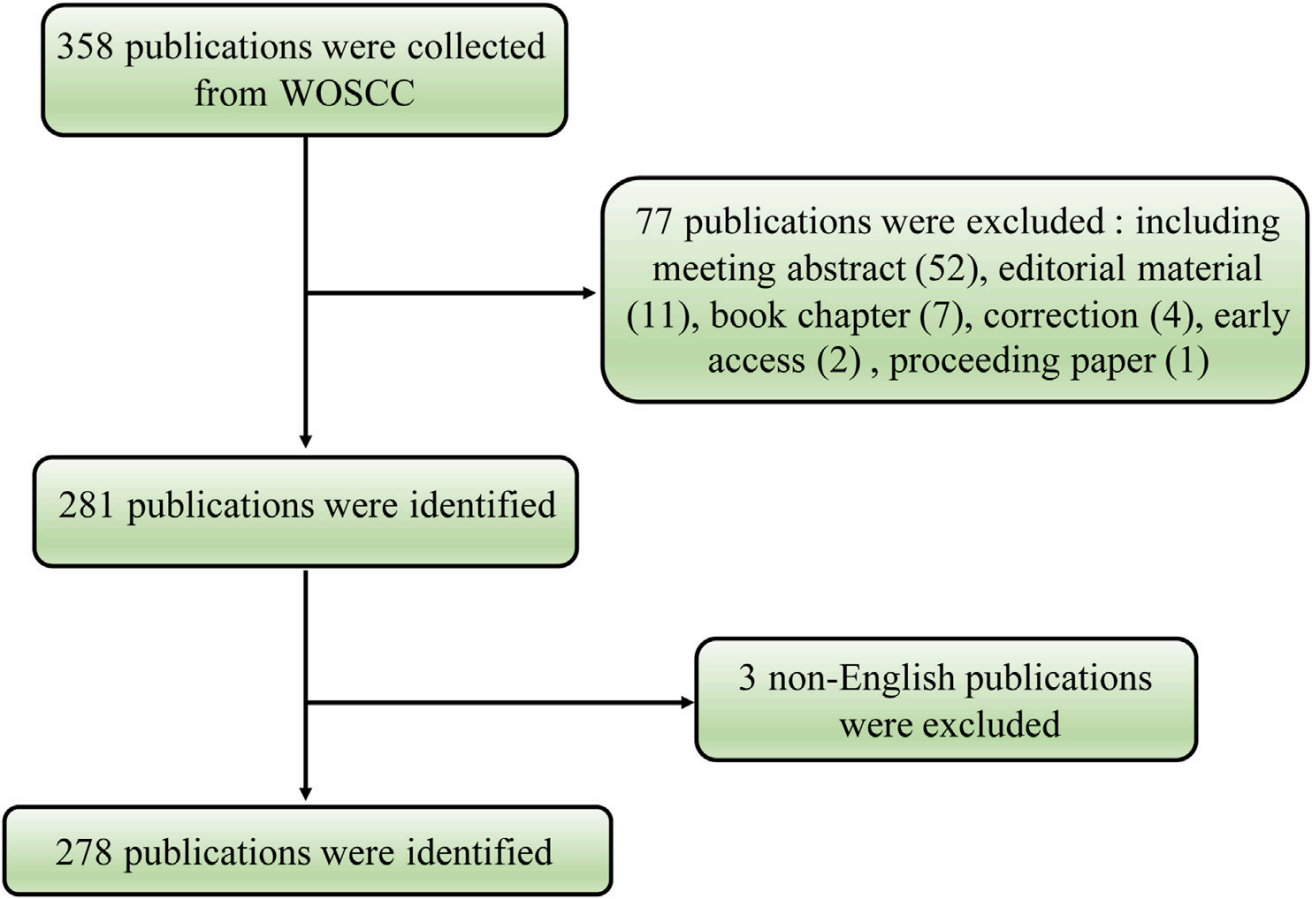
Flowchart of the volume-regulated anion channel (VRAC) research.

Initially, the Web of Science Core Collection (WoSCC) database yielded a total of 358 publications. The analysis included a total of 278 articles on the volume-regulated anion channel VRAC from 2014 to 2022, after excluding certain types of publications, including meeting abstracts (52), editorial material (11), book chapters (7), corrections (4), early access (2), proceeding paper (1), and three non-English articles in Chinese (1), Japanese (1) and Russian (1) (Figure 1).

**FIGURE 2:**
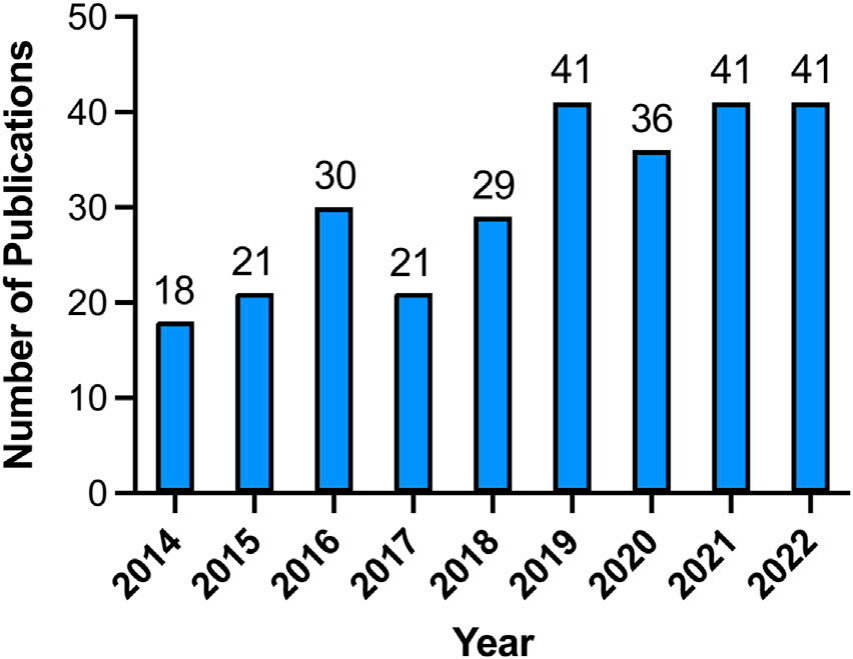
The yearly distribution of publications in the field of VRAC research from 2014 to 2022.

To examine the trends in VRAC research, we visualized the yearly outputs of relevant articles using a histogram (Figure 2). The results revealed a consistent increase in the quantity of research papers on VRAC during the past 8 years, with an average annual publication count of 34.75. Notably, in the year 2019, 2021, and 2022, a total of 41 articles were published each year, which represents a doubling of the compared to 2014 when 18 articles were published. Despite a slight decrease in the number of publications in 2017, VRAC remains a hot research topic, as evidenced by the publication of over 29 articles in the subsequent years (Figure 2).

### 3.2 Contributions of countries and organizations

**FIGURE 3:**
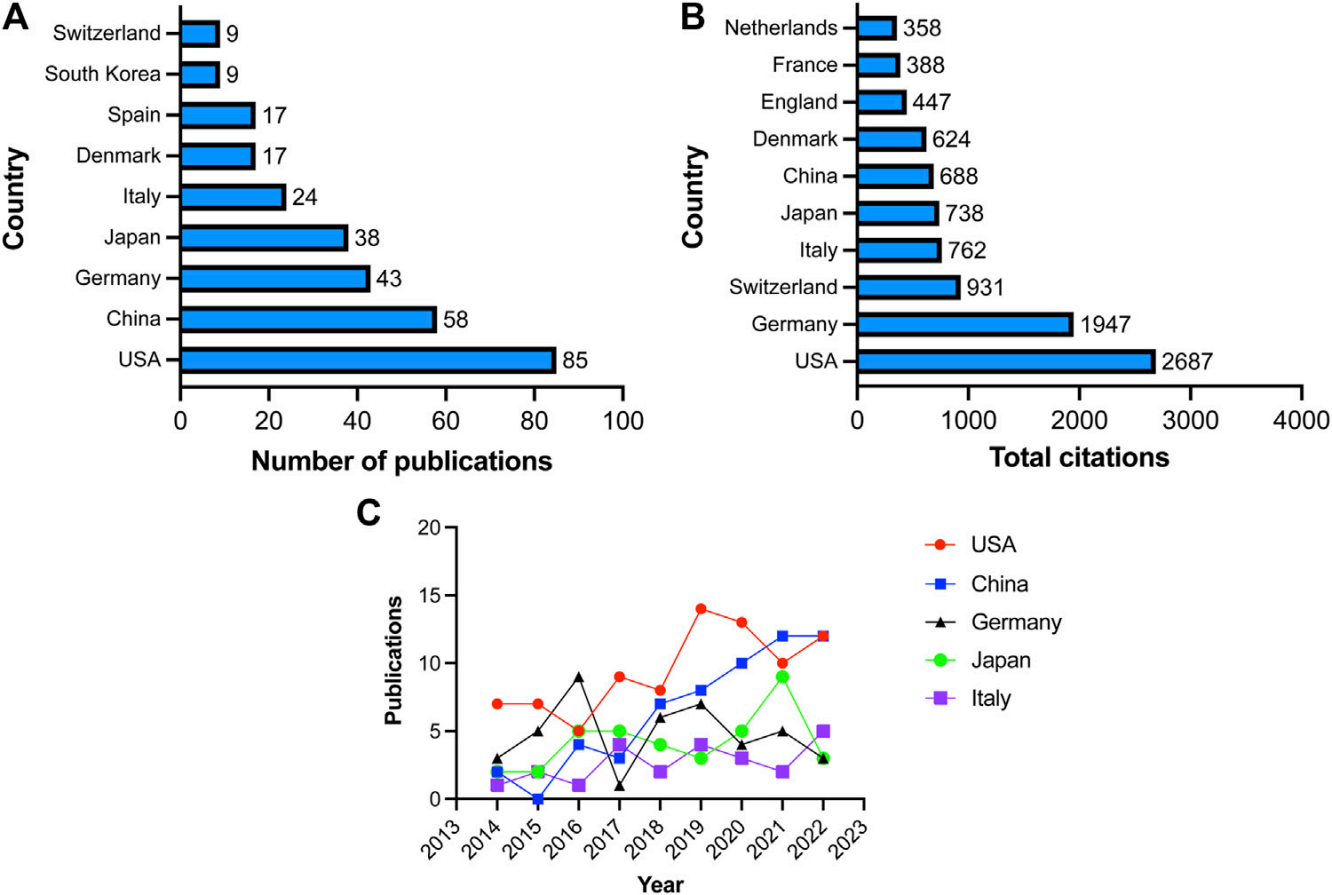
Countries involved in VRAC research. **(A)** Top 10 countries with the largest number of publications. **(B)** Total citations received by articles related to VARC from various countries. **(C)** Annual number of publications in United States, China, Germany, Japan and Italy.

Publications in the field were contributed by a total of forty-six countries and regions. Among them, the United States had the most publications with 85 articles, accounting for 30.58% of all articles. China followed closely with 58 publications (20.86%), followed by Germany with 43 publications (15.47%), Japan with 38 publications (13.67%), and Italy with 24 publications (8.63%). Other countries or regions contributed fewer than 20 articles (Figure 3A). Regarding the number of citations, publications originating from the United States garnered the most citations, totaling 2,687, followed by Germany with 1947 citations, Switzerland with 931 citations, Italy with 762 citations, and Japan with 738 citations (Figure 3B). We subsequently conducted a statistical analysis of the annual publication output for the top five countries based on publication number. As depicted in Figure 3C, the United States consistently ranking first in publication output, except for 2016 and 2021. The number of publications in China has exhibited a notable upward trend, increasing from 2 articles in 2014 to 12 articles in 2022. Germany initially experienced an increase in publication output, followed by a slight declining trend. Japan and Italy have relatively stable publication outputs, with no more than 5 papers published annually.

**FIGURE 4:**
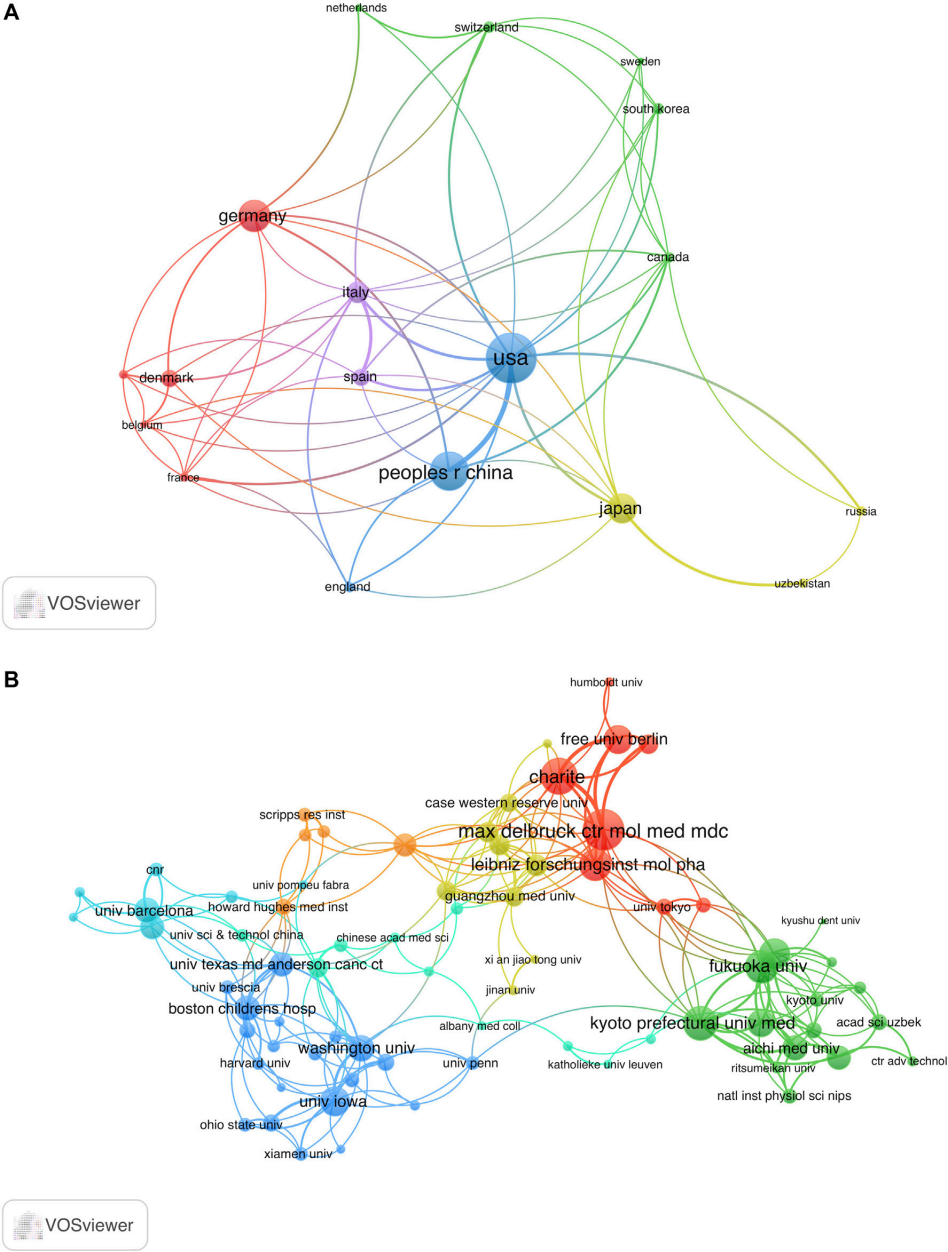
Analysis co-authorship of nations and organizations. **(A)** Co-authorship network map of countries with over three publications. The size of the node indicates the number of publications. **(B)** Co-authorship network map of institutions with over two publications. The size of the node represents total link strength. The thickness of the lines indicates the relationship strength.

The co-occurrence map can provide valuable insights and facilitate the collaborative relationships among researchers. In our analysis, we focused on 19 countries that had published more than three articles in the field, as shown in Figure 4A. Among these countries, the United States boasted the greatest overall link potency, with 56 connections. Italy followed with 28 connections, China with 26 connections, Japan with 19 connections, and Germany with 18 connections.

Research on VRAC engaged a total of 421 institutions. The University of Copenhagen emerged as the leading institution, contributing 16 publications, which account for 5.76% of all articles in this field. The Freie Universität Berlin ranked second with 15 publications (3.78%), followed by Kyoto Prefectural University of Medicine with 14 publications (2.52%), and Vanderbilt University with 11 publications (2.52%). Figure 4B depicts the co-authorship network involving 121 institutions with more than two papers to the VRAC research field. After eliminating 46 institutions with a total link strength below 2, we observed collaborative relationships among 75 institutions. Notably, the Max Delbrück Center for Molecular Medicine (MDC) emerged as the leading institution in terms of collaborative efforts, with the highest total link strength of 38. It is closely followed by Charité University Medicine Berlin with 31, Kyoto Prefectural University of Medicine with 30, Fukuoka University with 26, and the Leibniz-Forschungsinstitut für Molekulare Pharmakologie with 25.

### 3.3 Journals and research fields

Articles related to VRAC research were distributed across 149 journals. Among these, the top 10 most accounting for 30.22% of the total articles in the field (Table 1). Pflügers Archiv European Journal of Physiology emerged as the most popular journal for publishing articles on the VRAC channel, with 16 records, accounting for 5.76% of all articles. Following closely behind was the International Journal of Molecular Sciences with 11 publications, representing 3.96% of the total articles. Other prominent journals in the field included Frontiers in Cell and Developmental Biology, American Journal of Physiology Cell Physiology, and Channels, each with 9, 8, and 8 publications, respectively, contributing significantly to the dissemination of VRAC research findings.

**TABLE 1:** Top ten journals by number of VRAC-related articles.

Rank	Journals	Records (n)	Impact factor (2021)	JCR partition (2021)
1	Pflügers Archiv European Journal of Physiology	16	4.458	Q1/Q2
2	International journal of molecular sciences	11	6.208	Q1/Q2
3	Frontiers in cell and developmental biology	9	6.081	Q3/Q4
4	American journal of physiology cell physiology	8	5.282	Q1/Q2
4	Channels	8	3.493	Q3
6	eLife	7	8.713	Q1
6	Journal of Physiology-London	7	6.228	Q1
6	Nature communications	7	17.694	Q1
9	Journal of Biological Chemistry	6	5.486	Q1/Q2
10	Biochemical and Biophysical Research Communications	5	3.322	Q3

**FIGURE 5:**
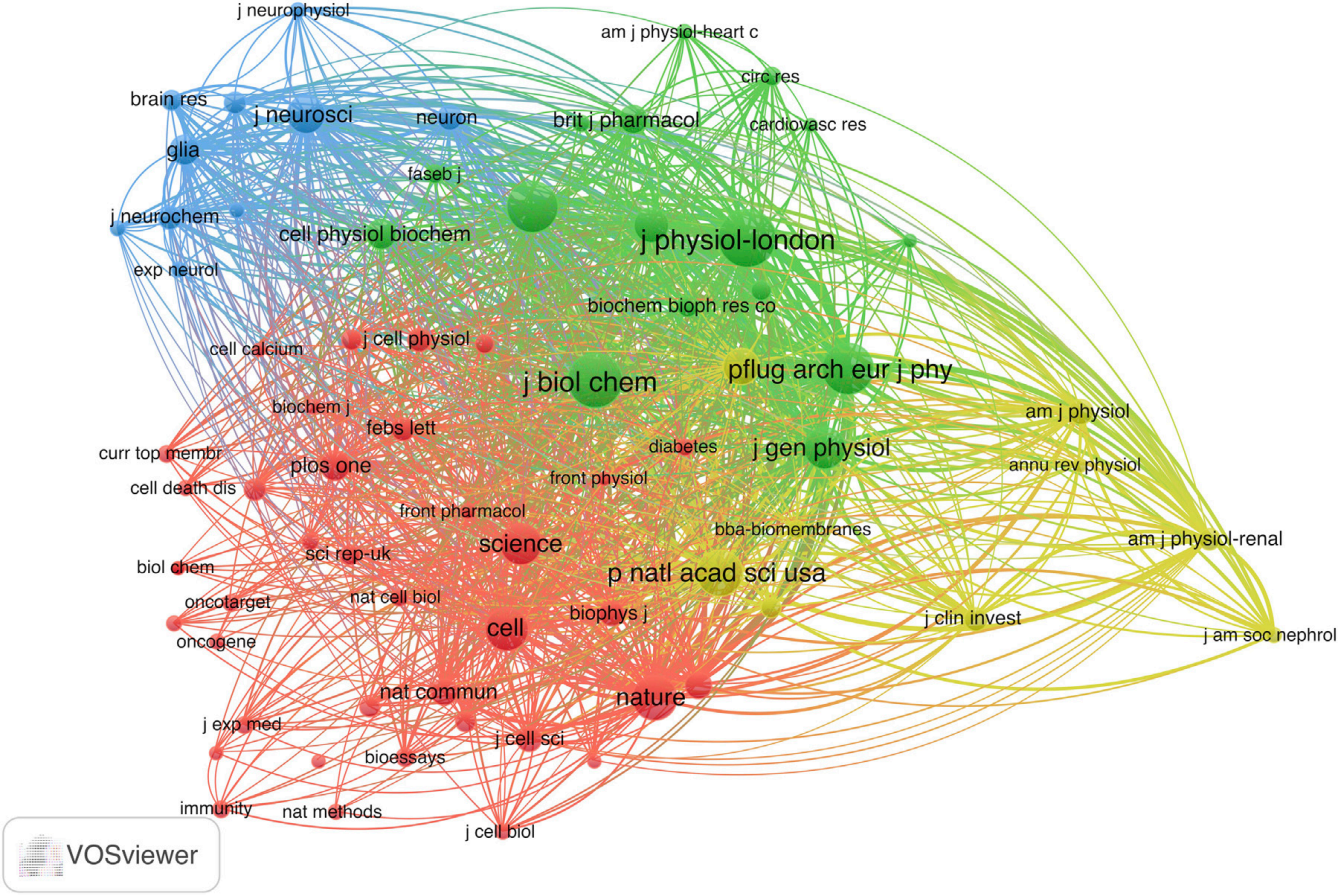
Co-citation analysis map of the journal patterns in VRAC research with over 50 documents. The size of the node indicates citations received by the journal.

Next, we conducted an analysis of 72 journals based on their co-citation frequency in over 50 publications (Figure 5). Table 2 presents ten journals that have published research related to VRAC and have received the most citations. The Journal of Physiology-London was cited the most with 853 citations, followed by the Journal of Biological Chemistry (771 citations), Pflügers Archiv European Journal of Physiology (686 citations), American Journal of Physiology-Cell Physiology (683 citations), Proceedings of the National Academy of Sciences (553 citations), Nature (502 citations). The others had less than 500 citations. Notably, 7 of 10 were categorized in the first quartile (Q1) only, and three boasted an impact factor of >60.

**TABLE 2:** Top ten journals by citations of VRAC-related articles.

Rank	Journals	Citations (n)	Impact factor (2021)	2021 JCR partition (2021)
1	Journal of Physiology-London	853	6.228	Q1
2	Journal of Biological Chemistry	771	5.486	Q1/Q2
3	Pflügers Archiv European Journal of Physiology	686	4.458	Q1/Q2
4	American Journal of Physiology-Cell Physiology	683	5.282	Q1/Q2
5	Proceedings of the National Academy of Sciences (PNAS)	553	12.779	Q1
6	Nature	502	69.504	Q1
7	Cell	475	66.850	Q1
8	Science	447	63.714	Q1
9	Journal of General Physiology	440	4.000	Q1
10	Journal of Neuroscience	361	6.709	Q1

The articles we identified in this analysis were categorized into 44 research areas. Biochemistry Molecular Biology was the research area with the highest representation, accounting for 70 records, which is equivalent to 25.18% of all articles. Following closely behind was Biochemistry Molecular Biology (70 records, 25.18% of all articles), followed by Cell Biology (57, 20.50%), Physiology (50, 17.99%), Neurosciences (37, 13.31%), and Pharmacology Pharmacy (27, 9.71%) (Table 3).

**TABLE 3:** Top ten widely explored and extensively researched fields on VRAC.

Rank	Research areas	Records (n)	% (of 278)
1	Biochemistry Molecular Biology	70	25.18
2	Cell Biology	57	20.50
3	Physiology	50	17.99
4	Neurosciences	37	13.31
5	Pharmacology Pharmacy	27	9.71
6	Multidisciplinary Sciences	22	7.91
7	Biology	15	5.40
8	Chemistry Multidisciplinary	14	5.04
9	Oncology	14	5.04
10	Biophysics	12	4.32

### 3.4 Analysis of authors

Thomas J. Jentsch emerged as the most prolific author in terms of publication productivity, with 12 articles (4.32% of all publications). Following closely behind were Rajan Sah (11, 3.96%), Yasunobu Okada and Tobias Stauber (each 10, 3.60%), and Karl Kunzelmann (9, 3.24%) (Table 4). When it comes to citations, Thomas J. Jentsch garnered the highest count (1,146 citations), followed by Florian Ullrich (876 citations), Zhaozhu Qiu (769 citations), Tobias Stauber (728 citations), and Darius Lutter (708 citations) (Table 4). Publications from Thomas J. Jentsch obtained the highest h-index of 11. Following closely behind were Rajan Sah and Tobias Stauber, both with an h-index of 9, Karl Kunzelmann and Raul Estevez achieved h-index of 8 (Table 4).

**TABLE 4:** Top 14 authors by number of publications related to VRAC research.

Rank	Authors	Records (n)	Total citations	Average citations	H-index
1	Thomas J. Jentsch	12	1,146	95.50	11
2	Rajan Sah	11	301	27.36	9
3	Tobias Stauber	10	728	72.80	7
3	Yasunobu Okada	10	248	24.80	9
5	Karl Kunzelmann	9	347	38.56	8
6	Alexander A. Mongin	8	298	37.25	8
6	Raul Estevez	8	113	14.13	5
6	Ravshan Z. Sabirov	8	104	13.00	7
9	Florian Ullrich	7	876	125.14	7
9	Rainer Schreiber	7	231	33.00	7
9	Ian Henry Lambert	7	190	27.14	5
9	Hector Gaitan-Penas	7	175	25.00	5
9	Tomohiro Numata	7	116	16.57	6
9	Yoshinori Marunaka	7	90	12.86	7

**FIGURE 6:**
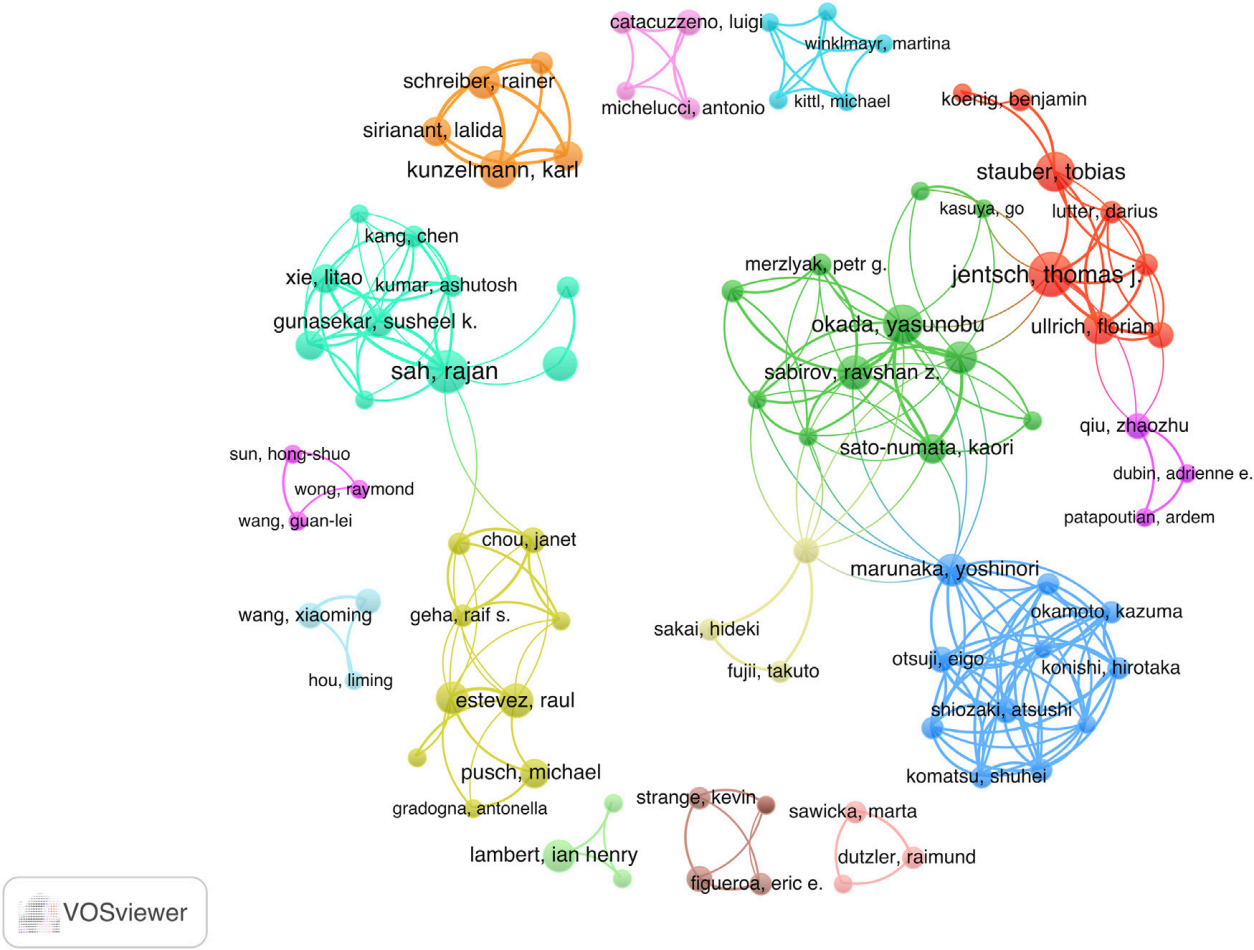
Visual map of co-authorship between authors with a minimum of 3 published articles. The size of the node indicates the counts of articles.

Next, we examined a network of 101 authors who collaborated on over three publications (Figure 6). The size of each node in the network is proportional to the quantity of publications, while the links between nodes illustrate the cooperative relationships among authors.

### 3.5 Analysis of citations and co-citations

**FIGURE 7:**
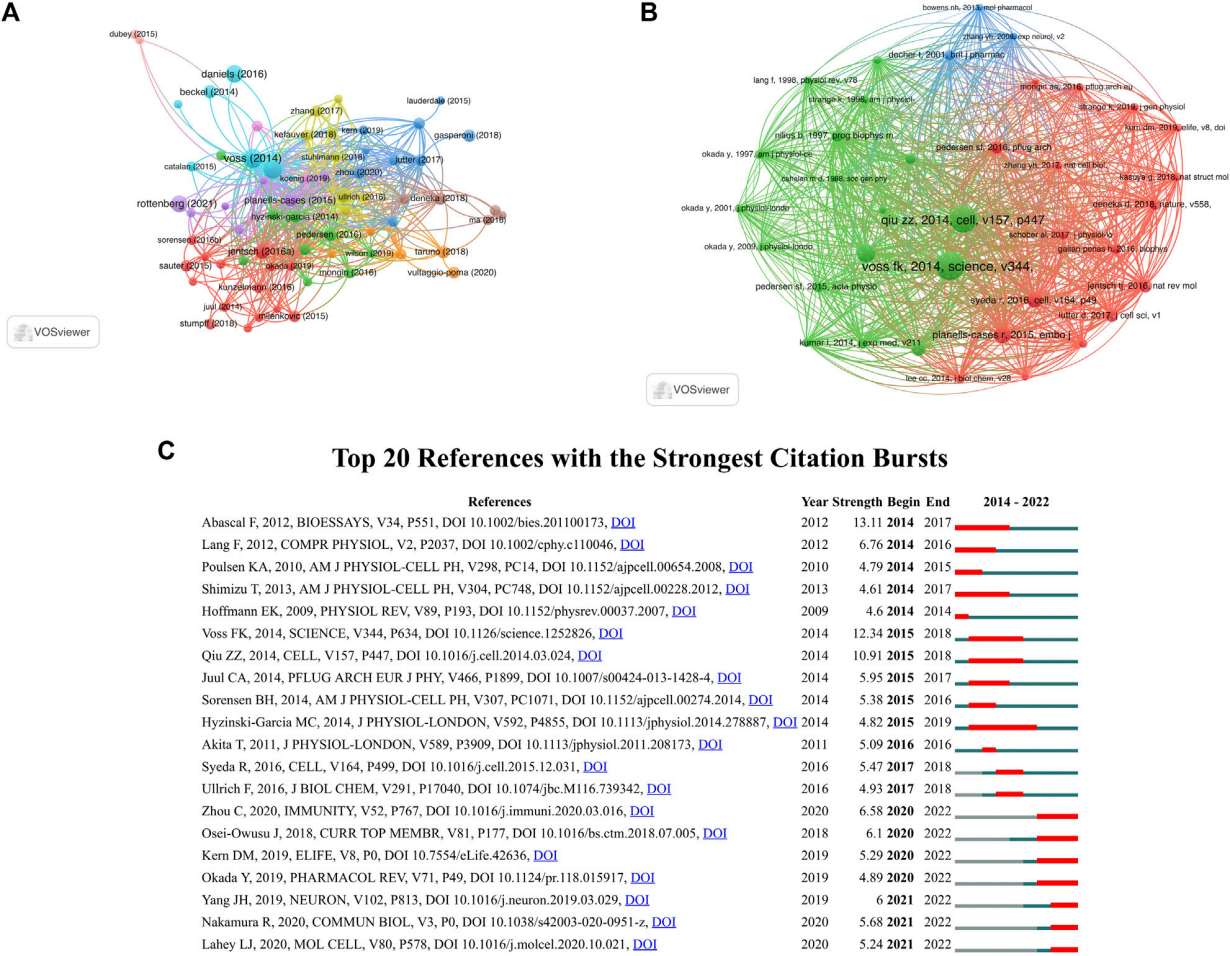
**(A)** Citation analysis network map of documents with no less than 30 citations. The node size represents the count of citations. **(B)** Co-citation analysis network map of references with no less than 30 citations. The size of the node represents the number of citations. **(C)** Top 20 references with the strongest citation bursts.

Figure 7A illustrated that VRAC research analysis revealed 72 documents that received more than 30 citations. The top ten highly cited documents are displayed in Table 5. Among them, “Identification of LRRC8 Heteromers as an Essential Component of the Volume-Regulated Anion Channel VRAC” by (Voss et al., 2014) had most citations (389), followed by “SWELL1, a Plasma Membrane Protein, Is an Essential Component of Volume-Regulated Anion Channel” by (Qiu et al., 2014), which had 366 citations. The article ranked third in terms of citations was “Fenamate NSAIDs inhibit the NLRP3 inflammasome and protect against Alzheimer’s disease in rodent models” by (Daniels et al., 2016), with 263 citations.

**TABLE 5:** Top ten most cited articles related to VRAC research.

Rank	Title	First author	Journal	Year	Citations (n)
1	Identification of LRRC8 Heteromers as an Essential Component of the Volume-Regulated Anion Channel VRAC	Felizia K Voss	*Science*	2014	389
2	SWELL1, a Plasma Membrane Protein, Is an Essential Component of Volume-Regulated Anion Channel	Zhaozhu Qiu	*Cell*	2014	366
3	Fenamate NSAIDs inhibit the NLRP3 inflammasome and protect against Alzheimer’s disease in rodent models	Michael J D Daniels	*Nature communications*	2016	263
4	The rediscovery of platinum-based cancer therapy	Sven Rottenberg	*Nature reviews cancer*	2021	239
5	VRACs and other ion channels and transporters in the regulation of cell volume and beyond	Thomas J Jentsch	*Nature review molecular cell biology*	2016	177
6	Subunit composition of VRAC channels determines substrate specificity and cellular resistance to Pt-based anti-cancer drugs	Rosa Planells-Cases	*EMBO Journal*	2015	166
7	LRRC8 Proteins Form Volume-Regulated Anion Channels that Sense Ionic Strength	Ruhma Syeda	*Cell*	2016	150
8	VRACs and other ion channels and transporters in the regulation of cell volume and beyond	Jonathan M Beckel	*Glia*	2014	124
9	Structure of a volume-regulated anion channel of the LRRC8 family	Dawid Deneka	*Nature*	2018	107
10	ATP Release Channels	Akiyuki Taruno	*International journal of molecular sciences*	2018	105

37 references were found to be co-cited in a minimum 30 citations (Figure 7B). As the co-citation analysis also takes those articles into account that were cited in the 278 papers on the selected VRAC between 2014–2022, it contains papers published before 2014. Table 6 presents the top ten co-cited documents, with the five most cited documents being Voss et al. (2014, Science; 199 citations), Qiu et al. (2014, Cell; 188 citations), Hoffmann et al. (2009, Physiological Reviews; 113 citations), Planells-Cases et al. (2015, EMBO J, 104 citations), and Syeda et al. (2016, Cell; 88 citations).

**TABLE 6:** Top ten co-cited papers in VRAC research field.

Rank	Title	First author	Journal	Publication Year	Citations (n)
1	Identification of LRRC8 Heteromers as an Essential Component of the Volume-Regulated Anion Channel VRAC	Felizia K Voss	*Science*	2014	199
2	SWELL1, a Plasma Membrane Protein, Is an Essential Component of Volume-Regulated Anion Channel	Zhaozhu Qiu	*Cell*	2014	188
3	Physiology of cell volume regulation in vertebrates	Else K Hoffmann	*Physiological Reviews*	2009	113
4	Subunit composition of VRAC channels determines substrate specificity and cellular resistance to Pt-based anti-cancer drugs	Rosa Planells-Cases	*EMBO Journal*	2015	104
5	LRRC8 Proteins Form Volume-Regulated Anion Channels that Sense Ionic Strength	Ruhma Syeda	*Cell*	2016	88
6	LRRC8s revisited: And now they SWELL!	Ancha Baranova	*Bioessays*	2014	76
7	Biophysics and Physiology of the Volume-Regulated Anion Channel (VRAC)/Volume-Sensitive Outwardly Rectifying Anion Channel (VSOR)	Stine F Pedersen	*Glia*	2014	68
8	VRACs and other ion channels and transporters in the regulation of cell volume and beyond	Thomas J Jentsch	*Nature review molecular cell biology*	2016	67
9	Selective transport of neurotransmitters and modulators by distinct volume-regulated LRRC8 anion channels	Darius Lutter	*Journal of cell science*	2017	63
10	Properties of volume-regulated anion channels in mammalian cells	Bernd Nilius	*Progress in Biophysics and Molecular Biology*	1997	63

Burst detection enables the identification of articles that receive significant attention from the scientific community during a specific time period. Essentially, a burst indicates that a publication has garnered exceptional levels of interest from its respective scientific community. Figure 7C displays the top 20 documents that exhibited the strongest citation bursts. “LRRC8 proteins share a common ancestor with pannexins, and may form hexameric channels involved in cell-cell communication” had the strongest burst (13.11) (Abascal and Zardoya, 2012). The article “LRRC8A protein is indispensable for swelling-activated and ATP-induced release of excitatory amino acids in rat astrocytes” authored by Hyzinski-Garcia et al. (Hyzinski-García et al., 2014), exhibited a burst period that extended from 2015 to 2019, making it the longest burst observed in the analysis.

### 3.6 Analysis of keyword co-occurrence

**FIGURE 8:**
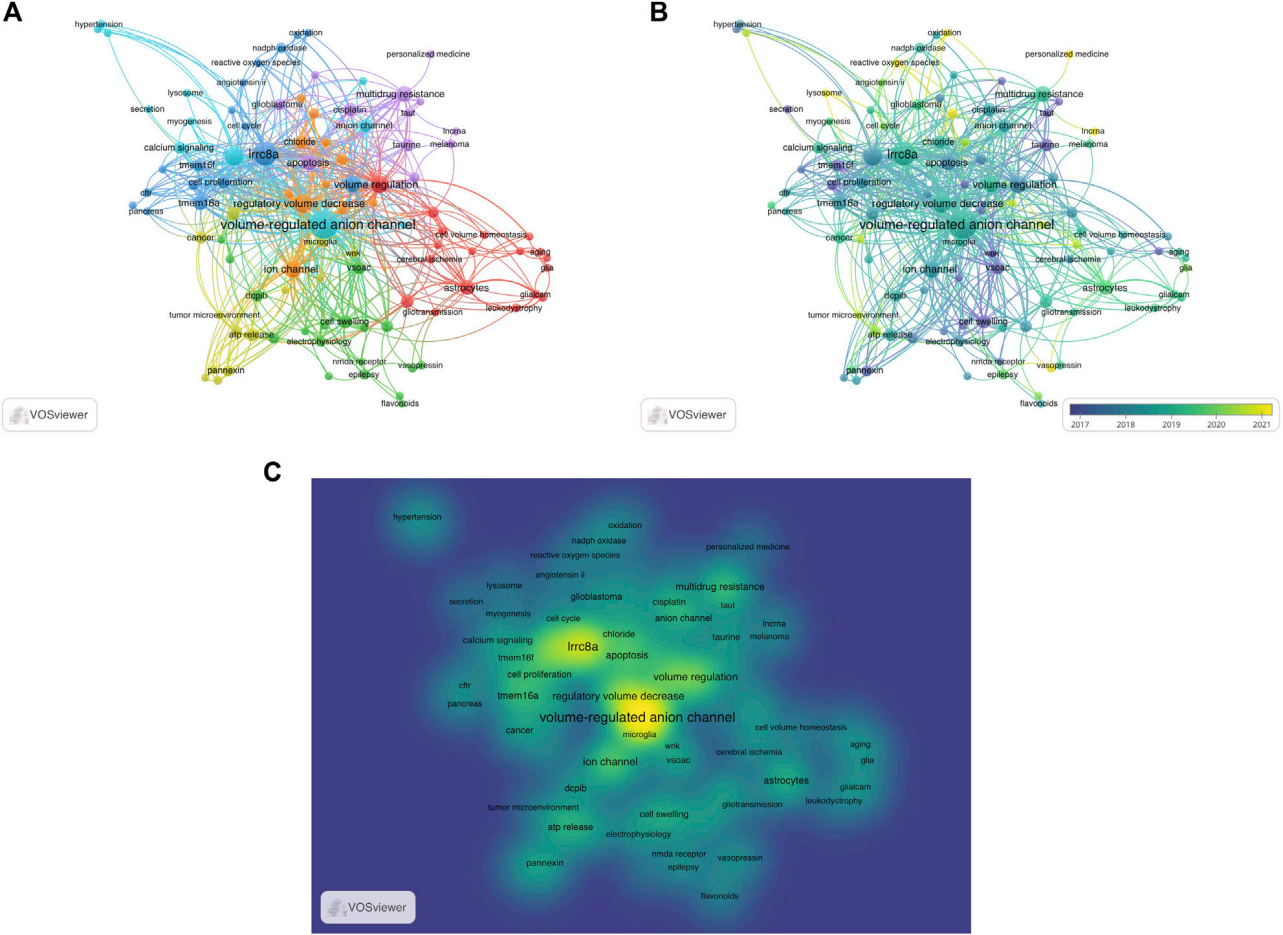
Analysis of keyword co-occurrence. **(A)** Keywords of studies are mapped. The more times the keyword appears, the larger its weight value is, which is depicted as size of the node and font. A shorter distance between two nodes generally signifies a stronger correlation, indicating the strength of the relationship between two items. A thicker line between two nodes indicates a closer relationship between two keywords that have a common occurrence. Different colors indicated the variety of clusters. **(B)** The distribution of keywords is categorized based on the average year of publication, with darker colors indicate earlier appearances in publications, lighter colors indicate more recent appearances. **(C)** The mean frequency of appearance determines the distribution of keywords, with yellow representing the highest frequency.

The utilization of keyword co-occurrence analysis enables the identification of emerging research patterns and focal points within a particular research field, offering valuable support for scientific research. In this study, 737 keywords were extracted from the 278 selected articles, yielding an average of approximately 2.65 keywords per article. Among these, 106 were found to occur more than two times (Figure 8A). The keyword with the highest frequency was “volume-regulated anion channel” with 84 occurrences. Other frequently keywords included “lrrc8a” (46), “chloride channel” (26), “regulatory volume decrease” (23), and “volume regulation” (21).

In Figure 8B, a visualization is shown that displays these keywords and colors them based on their average publication year. The more green or yellow hues depicts more recent usage and hence hints towards new advancements in VRAC research. The VRAC research focus has developed from the molecular identification and basic understanding of its role in regulating cell volume (keywords cell swelling (2017) and cell proliferation (2017)) to investigating its activation mechanisms (keywords such as oxidation (2021), reactive oxygen species (2021)), pharmacology (keywords such as flavonoids (2020), personalized medicine (2021)), and involvement in various physiological and pathological processes beyond osmotic volume regulation (keywords like glioblastoma (2019), astrocytes (2020), tumor microenvironment (2020), lysosome (2021), lncRNA (2021), and stroke (2021)).

Figure 8C, a density visualization, displays the identified keywords based on their frequency of appearance. Keywords in yellow indicate higher frequencies, while those in green appear less frequently. Density views are particularly beneficial for comprehending the overall structure of a map and highlighting the most significant areas. From Figure 8C, it is evident that “volume-regulated anion channel”, “lrrc8a”, and “volume regulation” are of particular importance in VRAC research, representing the core keywords of the field.

**FIGURE 9:**
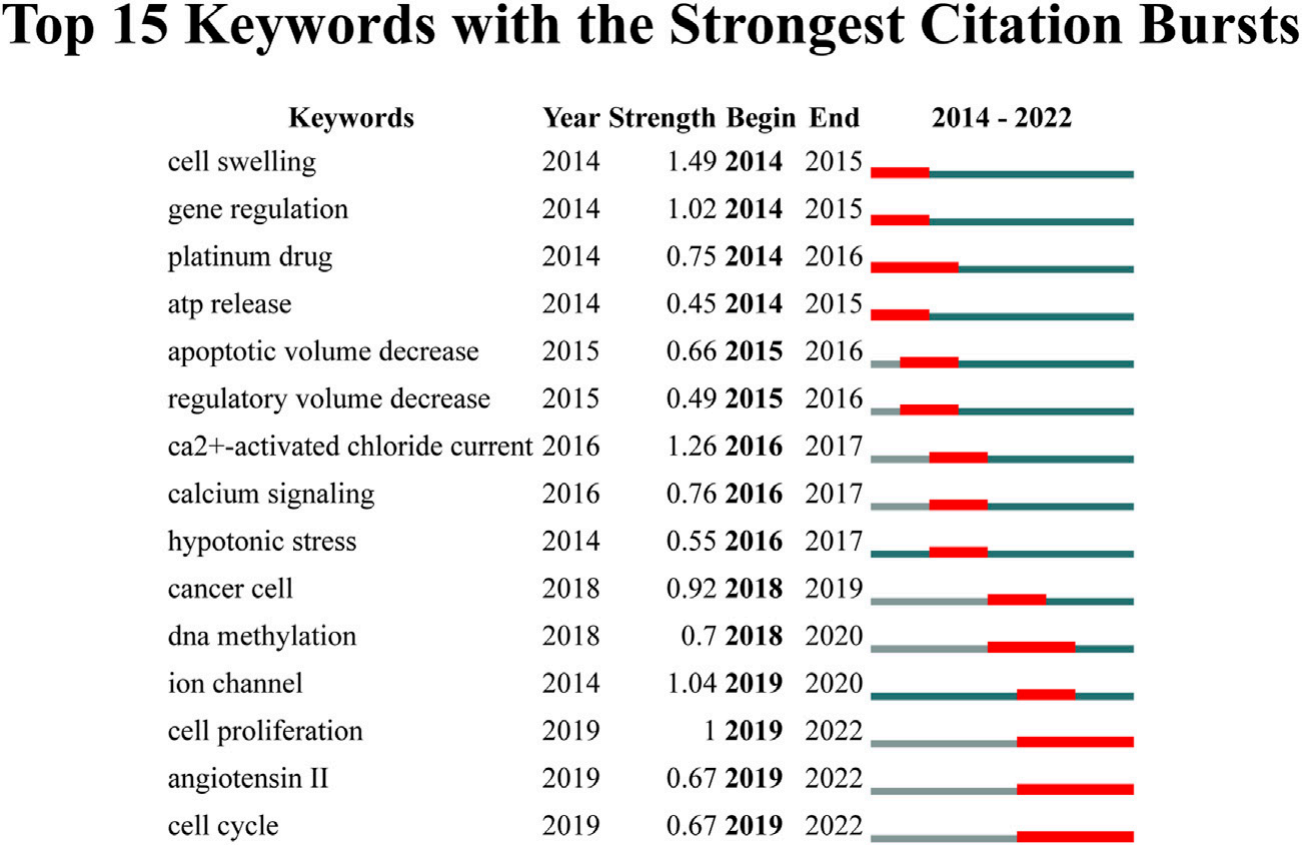
The top 15 keywords exhibiting the strongest citation bursts.

Furthermore, we used keyword bursts to reveal research directions in VRAC research field. In Figure 9, the top 15 keywords are depicted, which have the most citations. The keyword with the highest burst intensity were “cell swelling” (1.49), “ca2+-activated chloride current” (1.26), “ion channel” (1.04), “gene regulation” (1.02) and “cell proliferation” (1.00). The keyword with the longest duration was “cell proliferation”, “angiotensin II, and “cell cycle” (2019–2022).

## 4 Discussion

### 4.1 Overall patterns in VRAC research

VRAC-mediated chloride currents have been first described in 1988 (Cahalan and Lewis, 1988; Hazama and Okada, 1988), and subsequent biophysical and cell physiological studies were conducted by several laboratories throughout the 1990s. During this period, various molecular candidates for VRAC were proposed. However, none of these candidates were later proven experimentally to form VRAC (Wine and Luckie, 1996; Strange, 1998; Pu et al., 1999; Stobrawa et al., 2001; Nilius and Droogmans, 2003; Pedersen et al., 2015; Stauber, 2015). A significant breakthrough occurred in 2014 when two independently studies, published almost simultaneously, identified the genes responsible for VRAC through genome-wide RNA silencing screens in high-throughput fluorescence-based assays for VRAC function (Qiu et al., 2014; Voss et al., 2014).

In this study, we employed a combination of bibliometric analyses and network visualizations to provide an overview of the current state of VRAC channel research following its molecular identification in 2014. Our analysis encompassed various aspects, such as the involvement of nations, institutions, authors, and journals in this emerging area. By utilizing these analytical techniques, we aimed to not only describe the existing landscape but also predict the future research interests and hot topics that are likely to continue garnering attention in the VRAC field. This comprehensive approach allows for a deeper understanding of the research trends and dynamics within the VRAC research community, facilitating insights into the direction and potential areas of focus for future investigations.

Typically, the quantity of publications in a specific field serve as a reflection the scientific activities. In the case of VRAC research, we found that 1,501 authors affiliated with 421 institutions in 46 countries or regions have collectively contributed 278 publications from 2014 to 2022. Among these countries, the United States boasted the greatest quantity of publications and citations. Additionally, it maintains the top rank in country-based co-authorship, indicating its significant contribution and collaborative efforts in advancing the field. Over the past 8 years, there has been a significant rise in the number of studies carried out in China, Germany, Japan, and Italy over the past 8 years. Collectively, these countries accounted for 58.6% of all retrieved studies. Germany specifically ranked third in terms of total articles and second in total citations, and ranked fifth in terms of in cooperation with other countries. Furthermore, 421 institutions contributed articles to VRAC research. Among them, the University of Copenhagen from Denmark as the most prolific institution, with 16 published papers related to VRAC.

### 4.2 Influential authors and publications in VRAC research

Thomas J. Jentsch of the Max Delbrück Center for Molecular Medicine and the Leibniz-Forschungsinstitut für Molekulare Pharmakologie in Berlin, Germany, holds the records for the highest number of publications and citations. His research team is concerned with ion transport in the broadest sense. Their current endeavors revolve around unraveling the intricate relationship between structure and function in VRAC-LRRC8 proteins, aiming to elucidate their roles in different biological processes and diseases. After the molecular identification of VRAC (Voss et al., 2014), his group has demonstrated that VRAC participates in a wide array of processes, including resistance to cancer drugs (Planells-Cases et al., 2015), secretion of insulin by pancreatic β-cells (Stuhlmann et al., 2018), enhancing innate immunity by transporting the messenger molecule cGAMP (Zhou C. et al., 2020) and proximal tubules function and integrity (López-Cayuqueo et al., 2022). It has also contributed with studies on the structure-function relationship of VRAC (Ullrich et al., 2016; Zhou et al., 2018). Simultaneously, his team explores several other directions, including CLC chloride channels (Jentsch and Pusch, 2018), and the recently identified acid-activated anion channel ASOR (Yang et al., 2019a; Ullrich et al., 2019).

In terms of citation analysis and co-cited of references, it has been revealed that the seminal works published by Voss et al. in Science (Voss et al., 2014) and Qiu et al. in Cell (Qiu et al., 2014) accumulated the highest number of citations, not only because they are the earliest studies in the investigated time frame. These two publications separately discovered the genes responsible for encoding VRAC. LRRC8A, an essential VRAC subunit, was identified in both studies (Qiu et al., 2014; Voss et al., 2014). Voss et al. additionally revealed that VRACs are heteromeric channels consisted of LRRC8A and at least one other member from the LRRC8 family (LRRC8B-E) (Voss et al., 2014), subsequently also confirmed by the Patapoutian lab (Syeda et al., 2016). Surprisingly, the excessive expression of functional LRRC8 heteromers does not lead to an elevation of VRAC currents beyond the levels observed in the endogenous state. In fact, the over-expression of LRRC8A alone has the effect of reducing VRAC activity (Qiu et al., 2014; Voss et al., 2014).

### 4.3 Research hotspots and Frontiers

Our analysis of co-occurrence networks, which were organized based on topic area or publication date, have provided insight into the current hotspots and potential future directions in VRAC research (Figure 8). According to our keyword co-occurrence analysis, VRAC is implicated in various physiological processes, including but not limited to cell growth, movement, programmed cell death, swelling, and myogenesis. The transport of anions and organic osmolytes such as glutamate, taurine and ATP is part of its function. VRAC has also exhibited connections with other ion channels, including TMEM16A, TMEM16F, pannexin, and CFTR. It has been associated with various diseases including epilepsy, leukodystrophy, atherosclerosis, hypertension, diabetes, cerebral edema and stroke. It has been connected to different types of cancer including gastric cancer, glioblastoma, hepatocellular carcinoma and it has been implicated in anti-tumor drug resistance by its role in cellular drug uptake. In addition to general questions regarding the biophysics and biochemistry of VRAC, research on VRAC will be on particular physiological processes, each of which will address specific questions about the respective role of VRAC. Additionally, the pharmacology of VRAC has been studied. Here, we just highlight some examples.

#### 4.3.1 Activation mechanism of VRAC

Despite extensive investigation, the mechanism responsible for activating VRAC is still obscure and has remained one of the main questions to be addressed on a structure-functional, biochemical, cell biological and physiological level. A number of studies proposed that VRAC gating could be influenced or triggered by different stimuli, molecules within the cell, second messengers, and pathways of signaling. It is worth noting that a significant amount of research on VRAC activation was carried out before the identification of its molecular components (Stauber, 2015; Pedersen et al., 2016; Strange et al., 2019; Bertelli et al., 2021). The identification of LRRC8 heteromers as essential constituents allows using an increasing repertoire of molecular biological tools (Qiu et al., 2014; Voss et al., 2014; Kolobkova et al., 2021). In particular, the determination of LRRC8 complexes structures (Deneka et al., 2018; Kasuya et al., 2018; Kefauver et al., 2018; Kern et al., 2019; Nakamura et al., 2020; Deneka et al., 2021; Kern et al., 2023; Rutz et al., 2023; Takahashi et al., 2023) and other studies on the subunit composition and structure-function relationships (Gaitán-Peñas et al., 2016; Ullrich et al., 2016; Lutter et al., 2017; Schober et al., 2017; Yamada and Strange, 2018; Zhou et al., 2018; Pervaiz et al., 2019; Yamada et al., 2021; Bertelli et al., 2022), will greatly contribute to the understanding of the VRAC activation mechanism(s).

Intracellular ionic strength was implicated in regulating VRAC activity already before the molecular identification of this channel (Strange et al., 2019). Syeda et al. demonstrated that reduced intracellular ionic strength activated reconstituted VRAC in lipid droplet bilayer (Syeda et al., 2016) and reduced ionic strength has subsequently been utilized to activate VRAC currents in various electrophysiological studies. Several observations hint towards a role of the leucin-rich repeat domains (LRRDs) in VRAC gating: increased basal channel activity when proteins are fused to the C-terminal LRRDs (Gaitán-Peñas et al., 2016), a rearrangement of these domains during channel activation as shown by FRET measurements (König et al., 2019) and activating or inhibiting effect of synthetic nanobodies binding to the LRRDs (Deneka et al., 2021). As the LRRDs contain a large amount of charged amino acids, it has been hypothesized that reduced ionic strength changes the electrostatic interaction between the LRRDs within channel complex, thereby leading to VRAC opening. However, the reduction of ionic strength required to activate VRAC is much greater than that expected upon hypotonic activation; therefore, it is improbable that low intracellular ionic intensity serves as the physiologically significant trigger (Strange et al., 2019). Moreover, VRAC can be activated regardless of alterations in ionic concentration or extracellular osmolarity, such as through the use of sphingosine-1-phosphate or cisplatin (Burow et al., 2015; Planells-Cases et al., 2015; Gradogna et al., 2017a). Consistently, using FRET sensor within an intra-complex for monitoring VRAC activity, König et al. found that VRAC activity was not strictly coupled to reduced intracellular ionic strength (König et al., 2019).

Phosphorylation events were also implicated in the cellular signal transduction to VRAC gating (Bertelli et al., 2021). A number of studies have examined the impact of the protein kinase C (PKC) family on VRAC activity, yielding partly contradicting results as to its modulating effects on VRAC activity. For example, some studies have demonstrated that conventional PKC isoforms, specifically PKCα and PKCβI contributed to VRAC activation which induced by ATP (Rudkouskaya et al., 2008). PKCα has been demonstrated to have a function in controlling cell volume during hypotonic swelling (Senju et al., 2015). However, in human glioblastoma (GBM) cells, PKC activity was found to have no effect on VRAC gating (Catacuzzeno et al., 2014).

Another event that has been proposed as an underlying factor in VRAC gating is oxidation (Bertelli et al., 2021; Friard et al., 2021). Gradogna et al. demonstrated that VRAC is directly regulated by oxidation, and this regulation is dependent on the composition LRRC8 subunits (Gradogna et al., 2017b). Specifically, oxidation suppressed the activity of LRRC8A/LRRC8C and LRRC8A/LRRC8D, while it strongly potentiated the activity of LRRC8A/LRRC8E heteromers through intracellular cysteines oxidation (Gradogna et al., 2017a). Bertelli and others have recently discovered that oxidation targets two cysteines, namely, C424 and C448, which are situated in the initial two leucine rich repeats of LRRC8E. In addition, it was found that the oxidation of LRRC8C’s initial methionine hinders the functioning of VRAC (Bertelli et al., 2022). Conversely, VRAC was in turn shown to affect the cellular reactive oxygen species (ROS) levels, possibly through the release of reduced glutathione (Friard et al., 2019).

#### 4.3.2 The (patho)-physiological role of VRAC in the brain

VRAC is an activated channel that responds to cellular swelling and involved in regulateing cell volume reduction in most vertebrate cells, including those found in the central nervous system (CNS). In the brain, the role of VRAC extends to both normal and abnormal states (Akita and Okada, 2014; Mongin, 2016; Elorza-Vidal et al., 2019). For instance, VRAC has been implicated in the modulation of cell volume changes in astrocytes observed in megalencephalic leukoencephalopathy with cysts (MLC), which is associated with mutations in MLC1 or GlialCAM. However, the expression and subcellular distribution of LRRC8A, remain unaffected in mice with disrupted MLC1 or GlialCAM (Bugiani et al., 2017; Elorza-Vidal et al., 2018).

Mogin et al., proposed that VRAC in astrocytes and microglia may have significant physiological and detrimental effect by releasing excitatory amino acids (EAAs) like glutamate and aspartate in both normal and pathological conditions (Mongin, 2016). The release of astrocytic EAAs is considered to promote physiological interactions between astrocytes and neurons (Akita and Okada, 2014; Mongin, 2016; Elorza-Vidal et al., 2019). However, during brain ischemia, the release of EAAs by astrocytes is believed to contributed to neuronal cell death via excitotoxicity (Mongin, 2016). VRAC was reported to regulate EAA release in cultured astrocytes under swelling-induced and isovolumetric conditions (Hyzinski-García et al., 2014; Schober et al., 2017). Different subunits of VRAC are responsible for the release of specific EAAs, with LRRC8D facilitating the efflux of uncharged osmolytes and the LRRC8C/E combination was found to be crucial for the conductance of glutamate and aspartate (Lutter et al., 2017; Schober et al., 2017).

Research utilizing LRRC8A conditional knockout mice have provided insights regarding the role of VRAC in astrocytes during brain injury. These mice showed significantly reduced the volume of brain infarct and improved the score of neurological severity in a stroke model known as temporal middle cerebral artery occlusion (tMCAO), indicating that VRAC channel in astrocytes is indeed participated in brain damage which induced by stroke *in vivo* (Yang et al., 2019b; Zhou J. J. et al., 2020). Moreover, Zhou et al. reported that during cerebral ischemia, there is an elevation in neuronal VRAC currents that dependent on LRRC8A. This enhanced VRAC activity promotes the influx of glutamatergic to hippocampal neurons, contributing to ischemic stroke-induced brain injury (Zhou J. J. et al., 2020). In addition, the Qiu group recently found that VRAC-mediated ATP release from microglia in spinal cord is a key determinant of neuropathic pain (Chu et al., 2023).

#### 4.3.3 VRAC may represent a novel cancer therapeutic target

Ion channel dysfunction is increasingly associated with cancer (Prevarskaya et al., 2018). Among them, VRAC has been suggested to play a role in regulating cell growth, movement and apoptosis of tumor cells (Fraser and Pardo, 2008; Arcangeli and Becchetti, 2015; Sirianant et al., 2016; Prevarskaya et al., 2018). Downregulation of LRRC8A subunit has been reported to suppress the growth or proliferation of different cancer cell types, including human glioblastoma cells (Rubino et al., 2018), colorectal carcinoma cells (Fujii et al., 2018), esophageal squamous cell carcinoma cells (Konishi et al., 2019), gastric cancer (Kurashima et al., 2021), colon cancer (Zhang et al., 2018; Zhang et al., 2023) and cervical cancer (Chen et al., 2023), while LRRC8A overexpression reportedly facilitated hepatocellular carcinoma cell growth (Lu et al., 2019). *In vivo* studies in nude mouse models have also shown that LRRC8A downregulation suppressed the proliferation of colon carcinoma, hepatocellular carcinoma, and cervical cancer (Zhang et al., 2018; Lu et al., 2019; Chen et al., 2023).

Cell migration occurs during the whole cascade of cancer development and is particularly critical during metastasis. Local changes in cell volume, driven by a variety of ion transporters or channels, have been linked with and may even propel cell migration (Stroka et al., 2014). VRAC has been suggested to be one of these ion channels. Indeed, while pharmacological or molecular biological inhibition of VRAC did not detectably impair migration of various cell types in a study using a wound-healing assay (Liu and Stauber, 2019), other studies reported VRAC influenced the motility of a wide range of cancer cells such as colorectal cancer (Fujii et al., 2018), glioblastoma (Rubino et al., 2018; Wong et al., 2018), human colon cancer (Zhang et al., 2018), hepatocellular carcinoma (Lu et al., 2019), pancreatic adenocarcinoma (Xu et al., 2022) and breast cancer (Zhang et al., 2022).

Cancer cells frequently acquire multidrug resistance. VRAC was shown to be mediate cellular internalization of platinum-containing chemotherapeutic agents such as cisplatin and carboplatin into cells. The ability of these drugs to pass through VRAC is dependent on the presence of LRRC8D. Interestingly, when either LRRC8A or LRRC8D is knocked out, cancer cells exhibit a higher tolerance towards these platinum-based medications (Planells-Cases et al., 2015). LRRC8 genes have been implicated in drug resistance and prognosis in various cancer types (Sørensen et al., 2016; Xu et al., 2020; Siemer et al., 2021; Widmer et al., 2022; Xu et al., 2022).

#### 4.3.4 Pharmacology of VRAC

Given the involvement of VRAC in various physiological and pathological processes, of which we have covered only a few, the investigation of compounds that modify its activity is an important research field. For example, besides cancer and ischemic conditions after stroke, VRAC modulators were recently reported to be beneficial for murine type 2 diabetes (Gunasekar et al., 2022).

DCPIB (4-(2-butyl-6,7-dichlor-2-cyclopentyl- indan-1-on-5-yl)oxybutyric acid) has been identified as a highly potent selective VRAC inhibitor (Decher et al., 2001). Numerous studies have demonstrated that DCPIB effectively restrain VRAC activity in various types of cells, including HCT116, HeLa, HEK293, *Xenopus* oocytes, calf bovine pulmonary artery endothelial cells, Guinea-pig atrial cardiomyocytes, rat pancreatic beta cells and mouse astrocytes (Decher et al., 2001; Harrigan et al., 2008; Bantel et al., 2009; Akita and Okada, 2011; Schlichter et al., 2011; Stott et al., 2014; Yamada et al., 2021; Figueroa and Denton, 2022). DCPIB distinguishes itself from other VRAC blockers owing to its exceptional specificity for the VRAC channel, selectively targeting it over other chloride channels such as Cystic Fibrosis Transmembrane Conductance Regulator (CFTR), Calcium-activated Chloride Channels (CaCCs), and Proton-Activated Chloride (PAC), CLCs and Maxi-Cl channels (Decher et al., 2001; Sabirov and Okada, 2005; Sabirov et al., 2016; Sato-Numata et al., 2016). However, it experienced off-target effects on various transporters and channels including glutamate transporter GLT-1, K_ir_ and K_2P_ potassium channels, connexin hemichannels, H^+^,K^+^-ATPase (Bowens et al., 2013; Fujii et al., 2015; Deng et al., 2016; Lv et al., 2019). Moreover, DCPIB has been found to trigger K_2P_ K^+^ channels, particularly TREK1, TREK2 and TRAAK (Minieri et al., 2013; Lv et al., 2019). Additionally, it has been demonstrated to induced the activation of BK K^+^ channels (Zuccolini et al., 2022).

To identify further modulators of VRACs, Figueroa et al. recently screened 1,184 FDA-approved drugs and found that the cysteinyl leukotriene receptor 1 (CysLT1R) antagonists pranlukast and zafirlukast blocked VRAC currents in HEK293 cells in a dose-dependent manner (Figueroa et al., 2019; Figueroa and Denton, 2022). Importantly, VRAC inhibition by pranlukast and zafirlukast was independent of their known target CysLT1R. Furthermore, Xue et al. demonstrated that both natural and synthetic flavonoids can inhibit endogenous VRAC currents in HEK293 and HUVEC cells (Xue et al., 2018). Although these compounds are not fully specific to VRAC, their discovery as blockers of VRAC activity provide a valuable chemical framework for the creation of improved and targeted VRAC inhibitors.

Zinc pyrithione (ZPT), commonly utilized as an antifouling agent and for addressing conditions like dandruff and other skin disorders, was described as an activator of VRAC (Figueroa and Denton, 2021). In addition to enhances the rate of VRAC currents caused by cell swelling, ZPT also potentiated those currents even without cell swelling (Figueroa and Denton, 2021). In the future, it is necessary to study the mechanism of ZPT in activating VRAC, which will provide valuable insights into comprehending the relationship between channel structure, function and regulatory mechanisms.

### 4.4 Advantages and limitations

Based on our current understanding, this study represents the initial bibliometric analysis examining patterns in VRAC channel research area. Our analysis encompassed literature retrieved from 2014 to 2022, utilizing the data obtained from WoS (Web of Science), which covered a substantial portion of articles within the VRAC research. We conducted an unbiased, objective and comprehensive manner, providing a clear overview of the current status of VRAC research. However, it is important to acknowledge the limitations of our analysis. Firstly, this study focused exclusively on original articles and reviews that were published from 2014 to 2022 and indexed in the WoS database. Consequently, the exclusion of other publications types such as books and conference abstracts may have limited the representativeness of our data. To enhance the comprehensiveness of future analysis, incorporating data from additional databases like PubMed, Embase, and Scopus could enhance the comprehensiveness of our analysis. Nevertheless, it is worth noting that WoSCC (Web of Science Core Collection) provides detailed information, including annual publications, journals, author information, and national and institutional details, thereby contributing to the robustness of our findings. Furthermore, our analysis was restricted to English-language studies, potentially limiting the global perspective of our findings. Nevertheless, it is important to recognize that the results of this study were stable and highly replicable. With the inclusion of a vast majority of papers published between 2014 and 2022, the latest published studies would not significantly impact the final results.

## Data Availability

The original contributions presented in the study are included in the article, further inquiries can be directed to the corresponding author.
